# Serum levels of HMW adiponectin and its receptors are associated with cytokine levels and clinical characteristics in chronic obstructive pulmonary disease

**DOI:** 10.1515/med-2024-0904

**Published:** 2024-02-05

**Authors:** Li Lu, Mengyu Cheng

**Affiliations:** Department of Endocrinology, Taiyuan People’s Hospital, Taiyuan, 030001, P.R. China; Department of Respiratory and Critical Care Medicine, Shanxi Bethune Hospital, Shanxi Academy of Medical Sciences, Tongji Shanxi Hospital, Third Hospital of Shanxi Medical University, Taiyuan, 030032, China; Department of Respiratory and Critical Care Medicine, Tongji Hospital, Tongji Medical College, Huazhong University of Science and Technology, Wuhan, 430030, China

**Keywords:** adiponectin, adiponectin receptor, chronic obstructive pulmonary disease, cytokines, high molecular weight

## Abstract

We aimed to investigate the changes in the levels of high-molecular-weight (HMW) adiponectin, adiponectin receptors, and cytokines in patients with chronic obstructive pulmonary disease (COPD), as well as their potential relationships. Forty-one patients who underwent lobectomy for lung lesions and had a clear postoperative pathological diagnosis were divided into the non-COPD (*N* = 23) and COPD (*N* = 18) groups. HMW adiponectin, cytokine, and T-cadherin levels in serum and tissues were detected by enzyme-linked immunosorbent assay. The levels of HMW adiponectin and cytokine (interleukin [IL]-6, IL-10, surfactant protein D, 4-hydroxynonenal, tumor necrosis factor-α, and C reactive protein) in the serum and tissues increased in the COPD group compared to those in the non-COPD group. Patients with COPD exhibited AdipoR1 upregulation and AdipoR2 downregulation. Although T-cadherin did not differ significantly between patients with and those without COPD, its expression was elevated during the progression from COPD with benign lung lesions to combined lung cancer. Furthermore, the HMW adiponectin levels were significantly correlated with the cytokine levels and the clinical characteristics of COPD. HMW adiponectin and its receptors affect the inflammatory process in COPD and may further contribute to the progression of the disease to malignancy.

## 1 Introduction

Chronic obstructive pulmonary disease (COPD) is a chronic disease caused by airway and alveolar abnormalities and is characterized by persistent respiratory symptoms and irreversible airflow limitation; its typical symptoms include breathlessness, cough, and/or expectoration [1,2]. Patients with COPD may have obstructive bronchiolitis, emphysema, or a combination of both [3]. COPD results from a complex interplay between respirable particle exposure (e.g., cigarette smoke, air pollutants, and occupational dust) and multiple host factors (e.g., genetic, developmental, and social factors) [4,5]. Owing to persistent smoking exposure and an aging population, COPD may become the third leading cause of fatality globally by 2030, increasing the burden of living for 400 million people [6,7].

Pathologically, COPD is closely related to chronic airway inflammation and lung parenchyma, typically manifested by increased infiltration of neutrophils, macrophages, and lymphocytes into the airways [8]. These inflammatory cells release various pro-inflammatory factors such as cytokines, chemokines, growth factors, and lipids, resulting in a progressive and irreversible decline in lung function [9]. However, the molecular basis of this persistent inflammatory response and amplifying effects in COPD is inconclusive, preventing anti-inflammatory therapy from being the first-line treatment strategy for COPD [10].

Adiponectin is a complement-associated protein of adipocytes that is secreted and extensively circulates in the serum as a complex of different molecular weights, with high-molecular-weight (HMW) oligomers being the most potent active form [11]. As a unique adipokine, adiponectin is involved in many cellular physiological processes, including energy metabolism, inflammation, and immune responses [12,13]. Adiponectin is a key interaction signal between the lungs and adipose tissue and may act as a potent anti-inflammatory molecule against lung injury [14]. Studies have shown that the serum adiponectin levels are elevated in patients with COPD and correlate with disease severity [15]. However, the effect of adiponectin upregulation on COPD remains controversial. An animal study confirmed that adiponectin knockout mice exhibit a progressive COPD phenotype with systemic inflammation [16]. In addition, adiponectin induces an activating program of inflammation in macrophages and CD4+ T cells [17]. Therefore, whether adiponectin plays an anti- or pro-inflammatory role in COPD remains unclear. Adiponectin is a promising candidate, and its serum level is a potential biomarker for predicting clinical outcomes in COPD and other complications, independent of the significant effects of the smoking status [18].

However, whether there are changes in the expression levels of adiponectin receptors and other cytokines in patients with COPD, and whether these changes are, in turn, potentially related to adiponectin and, thus, affect its biological function, has not been determined. Thus, in this study, we aimed to evaluate the changes in the expression of HMW adiponectin, adiponectin receptors, and inflammatory cytokines in COPD, as well as their potential links, using tissues and serum collected from patients with COPD. Our findings will further explain the role of HMW adiponectin and its receptors in the inflammatory response of patients with COPD and their clinical relevance.

## 2 Methods

### 2.1 Study populations

Forty-one patients (sex, 26 males and 15 females) who underwent lobectomy for lung lesions and had a clear postoperative pathological diagnosis were enrolled from 2013 to 2015 in the Department of Thoracic Surgery of the Shanxi Bethune Hospital. According to the diagnostic criteria for COPD (forced expiratory volume in 1 s [FEV1]/forced vital capacity ratio <0.70) [19], all participants were divided into the non-COPD (*N* = 23; age, 54.40 ± 13.08 years) and COPD (*N* = 18; age, 61.00 ± 5.437 years) groups. The non-COPD group included 13 patients with benign lesions (6, 4, and 3 patients with pulmonary bulla, inflammatory pseudotumor, and bronchiectasia, respectively) and 10 patients with non-small-cell lung cancer (NSCLC), whereas the COPD group included 10 patients with benign lesions (4, 4, and 2 patients with pulmonary bulla, bronchiectasia, and tuberculoma, respectively) and 8 patients with NSCLC. All patients had no symptoms of systemic or respiratory infection within 2 weeks prior to surgery and had normal blood leukocyte counts, blood sedimentation, or liver and kidney functions. The exclusion criteria included: (1) combination of other lung diseases such as tuberculosis, pneumonia, interstitial lung disease, bronchial asthma, and bronchiectasis; (2) hypertension, diabetes, coronary artery disease, and other systemic and infectious diseases; (3) obstructive pneumonia caused by various causes; (4) COPD in acute exacerbation; (5) preoperative radiotherapy or chemotherapy; and (6) unwillingness to cooperate and complete inability to communicate.


**Informed consent:** All participants provided signed informed consent.
**Ethical approval:** This study was approved by the Ethics Committee of Shanxi Medical University (approval number: 2013035).

### 2.2 Sample collection

Surgical plans of the patients were formulated and implemented by the thoracic surgeon. Preoperative clinical information, such as the patient’s respiratory symptoms, medication history, smoking history, and COPD assessment test (CAT) score [20], as well as test results of pulmonary function and blood gas analysis at 1 week prior to surgery, were recorded. After fasting overnight before surgery, the patient was given 10 mL of peripheral vein solution in the early morning, followed by centrifugation of their blood at 3,000 rpm for 10 min, and the serum was separated and stored in a −80°C refrigerator until use. Intraoperatively, lung tissues >5 cm away from the lung cancer lesion were collected, and fresh peripheral lung tissue without lung cancer infiltration was visualized by the naked eye, rinsed with phosphate-buffered saline, and stored at −80°C for backup.

### 2.3 Enzyme-linked immunosorbent assay (ELISA)

The concentration of HMW adiponectin, interleukin (IL)-6, IL-10, surfactant protein D (SP-D), 4-hydroxynonenal (4-HNE), tumor necrosis factor α (TNF-α), C-reactive protein (CRP), and T-cadherin in serum and tissues of patients were quantified by the corresponding procedures of ELISA kits, which were all supplied by Shanghai WesTang Biotechnology Co., Ltd (Shanghai, China). After the enzyme-linked reaction, the optical density of each sample was measured at 450 nm using a microplate reader (BioTek, Winooski, VT, USA) for colorimetric analysis.

### 2.4 Western blot

Lung tissue (50 mg) was added with 0.5 mL of the protein lysate (Cell Signaling Technology, Danvers, MA, USA), followed by sonication and homogenization. Then, samples were centrifuged at 10,000 × *g* for 10 min at 4°C to extract the supernatant for the determination of protein concentration (10 µg/µL) and western blotting. Electrophoresis, membrane transfer, and sealing were performed according to conventional procedures. Primary antibodies, including anti-adiponectin receptor 1 (AdipoR1) (ab126611, Abcam, Cambridge, UK), anti-AdipoR2 (ab77612, Abcam), and anti-T-cadherin (ab167407, Abcam), were diluted 1,000 times and incubated overnight. Anti-β-actin antibody (1:1,000, ab8226, Abcam) was used as an internal reference. Then, the membranes were incubated with the secondary antibody at a dilution of 1:10,000. The membranes were placed on a gel imager (Bio-Rad, Hercules, CA, USA) for development and gray-value calculations.

### 2.5 Statistical analysis

Statistical analysis was performed using SPSS version 17.0 software package (Chicago, IL, USA), and all experimental data are expressed as means ± standard deviations. The chi-square test or independent sample *T*-test was used for comparison between the two groups where appropriate. To control for the effect of NSCLC variables, covariance analysis was performed to compare the data of the non-COPD group to those of the COPD group. Spearman’s correlation analysis was used to assess the relationships among variables. Statistical significance was set at *P* < 0.05.

## 3 Results

### 3.1 Demographic features and clinical data of all participants

The demographic data and clinical indices of all study participants are presented in Table 1. The enrolled participants in the non-COPD and COPD groups were matched in age and sex, and there were no significant differences in body mass index and smoking index. Furthermore, in the COPD group, the levels of PaO_2_ and FEV1 significantly decreased, whereas the PaCO_2_ level and CAT scores were significantly elevated compared to those in the non-COPD group. These results were consistent with the clinical manifestations of COPD, indicating that the patients were grouped reasonably and accurately.

**Table 1:** Clinical characteristics of all participants

Variables	Non-COPD (*N* = 23)	COPD (*N* = 18)	*t*	*P*
Gender (male/female)	15/8	11/7	*χ* ^2^＝3.20	>0.05
Age (years)	54.40 ± 13.08	61.00 ± 5.44	−1.473	0.158
PaO_2_ (mmHg)	79.84 ± 4.35	66.08 ± 7.81	4.866	**0.001**
PaCO_2_ (mmHg)	39.13 ± 3.07	45.93 ± 2.48	−5.448	**0.001**
FEV1 (% predicted)	90.51 ± 11.13	70.70 ± 15.38	3.300	**0.004**
BMI (kg/m^2^)	23.47 ± 3.87	20.66 ± 2.94	1.831	0.085
CAT score	1.90 ± 3.21	13.7 ± 3.02	−8.462	**0.002**
Smoking index (pack-years)	480.00 ± 706.70	840.00 ± 751.60	−1.103	0.284

COPD: chronic obstructive pulmonary disease; PaO_2_: partial pressure of oxygen; PaCO_2_: partial pressure of carbon dioxide; FEV1: forced expiratory volume in 1 s; BMI: body mass index; CAT: COPD assessment test. Bold *P* < 0.05 indicates statistical significance.

### 3.2 Differences in levels of HMW adiponectin and cytokines between the patients with and without COPD

Using ELISA, the levels of HMW adiponectin and cytokines in both the serum and lung tissues of patients were detected and, then, compared between the non-COPD and COPD groups (Table 2). The concentrations of HMW adiponectin in the serum and tissues of patients with COPD were significantly higher than those of patients without COPD. Cytokines, including IL-6, IL-10, SP-D, 4-HNE, TNF-α, and CRP, tended to be significantly elevated in the serum and tissues of patients in the COPD group compared to those in the non-COPD group. The upregulation of these hormones and cytokines suggests a higher level of inflammation in COPD.

**Table 2:** Concentration difference in HMW adiponectin and cytokines between non-COPD and COPD patients

Variables	Non-COPD (*N* = 23)	COPD (*N* = 18)	*F*	*P*
HMW adiponectin (serum, µg/mL)	0.88 ± 0.47	0.94 ± 0.06	33.42	**0.004**
HMW adiponectin (tissue, µg/mL)	0.94 ± 0.07	0.99 ± 0.05	25.90	**0.003**
IL-6 (serum, pg/mL)	17.41 ± 3.46	20.78 ± 4.28	27.98	**0.003**
IL-6 (tissue, pg/mL)	18.42 ± 3.052	21.89 ± 3.41	91.44	**0.004**
IL-10 (serum, pg/mL)	3.00 ± 0.49	3.48 ± 0.44	30.78	**0.001**
IL-10 (tissue, pg/mL)	3.57 ± 0.48	3.94 ± 0.41	16.53	**0.002**
SP-D (serum, ng/mL)	66.49 ± 12.64	80.98 ± 10.18	65.94	**0.005**
SP-D (tissue, ng/mL)	74.10 ± 15.20	91.09 ± 11.27	86.27	**0.001**
4-HNE (serum, ng/mL)	221.39 ± 49.76	275.92 ± 49.84	45.49	**0.001**
4-HNE (tissue, ng/mL)	381.96 ± 56.79	530.33 ± 63.10	38.71	**0.004**
TNF-α (serum, pg/mL)	31.52 ± 5.16	36.18 ± 3.18	29.20	**0.002**
TNF-α (tissue, pg/mL)	35.32 ± 4.77	40.29 ± 5.11	32.94	**0.003**
CRP (serum, ng/mL)	193.99 ± 29.95	231.44 ± 41.54	38.32	**0.002**
CRP (tissue, ng/mL)	224.08 ± 46.69	287.99 ± 61.82	52.88	**0.004**

COPD: chronic obstructive pulmonary disease; HMW: high molecular weight; IL-6: interleukin-6; IL-10: interleukin-10; SP-D: surfactant protein D; 4-HNE: 4-hydroxynonenal; TNF-α: tumor necrosis factor α; CRP: C reactive protein. Bold *P* < 0.05 indicates statistical significance.

### 3.3 Expression differences in adiponectin receptors between the non-COPD and COPD groups

**Figure A1:**
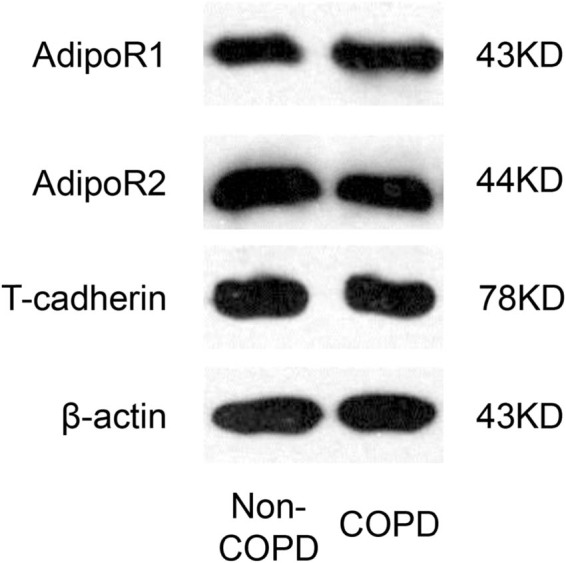
The original bands of adiponectin receptors including AdipoR1, AdipoR2, and T-cadherin in tissues of patients in the non-COPD and COPD groups detected by the western blot experiment. Abbreviations: AdipoR1, adiponectin receptor 1; AdipoR2, adiponectin receptor 2; COPD, chronic obstructive pulmonary disease.

The expression of AdipoR1, AdipoR2, and T-cadherin in the tissue, as well as T-cadherin in the serum, was detected by western blotting and ELISA, respectively. The bands in the western blot experiments are shown in Figure A1. When comparing the non-COPD and COPD groups, the protein expression level of AdipoR1 significantly increased, but AdipoR2 was significantly downregulated in patients with COPD (Table 3). However, no significant change was observed in T-cadherin expression in either tissue or serum in the COPD group compared to that in the non-COPD group (Table 3).

**Table 3:** Expression levels of adiponectin receptors in the non-COPD and COPD groups

Variables	AdipoR1	AdipoR2	T-cadherin (tissue)	T-cadherin (serum, ng/mL)
Non-COPD	145.70 ± 13.18	193.20 ± 10.57	143.20 ± 35.55	49.28 ± 3.87
COPD	167.50 ± 22.94	141.80 ± 9.01	147.80 ± 25.31	49.26 ± 3.53
*F*	8.050	23.440	0.805	0.001
*P*	**0.011**	**0.001**	0.108	0.982

COPD: chronic obstructive pulmonary disease; AdipoR1: adiponectin receptor 1; AdipoR2: adiponectin receptor 2. Bold *P* < 0.05 indicates statistical significance.

### 3.4 Correlations between HMW adiponectin with cytokines and clinical indexes in the COPD groups

To further investigate the relationships between HMW adiponectin and cytokines and the clinical features of COPD, Spearman’s correlation analysis was performed. The results suggest that the level of HMW adiponectin in patients with COPD exhibited significant positive correlations with serum cytokine levels (including IL-6, IL-10, CRP, TNF-α, 4-HNE, and SP-D) (Table 4). In addition, the serum levels of HMW adiponectin were significantly positively correlated with the CAT scores and negatively correlated with FEV1 and PaO_2_ (Table 4).

**Table 4:** Correlation analysis of serum HMW adiponectin with cytokines and clinical indicators in patients with COPD

Variables	IL-6	IL-10	CRP	TNF-α	4-HNE	SP-D	FEV1 (% predicted)	PaO_2_ (mmHg)	CAT score
*r*	0.621	0.463	0.455	0.392	0.431	0.247	−0.455	−0.313	0.466
*P*	**<0.05**	**<0.05**	**<0.05**	**<0.05**	**<0.05**	**<0.05**	**<0.05**	**<0.05**	**<0.05**

HMW: high molecular weight; COPD: chronic obstructive pulmonary disease; IL-6: interleukin-6; IL-10: interleukin-10; CRP: C reactive protein; TNF-α: tumor necrosis factor α; 4-HNE: 4-hydroxynonenal; SP-D: surfactant protein D; FEV1: forced expiratory volume in 1 s; PaO2: partial pressure of oxygen; CAT: COPD assessment test. Bold *P* < 0.05 indicates statistical significance.

### 3.5 Differences in HMW adiponectin, cytokines, and adiponectin receptor levels in serum and tissues between patients with COPD with and without NSCLC

**Figure A2:**
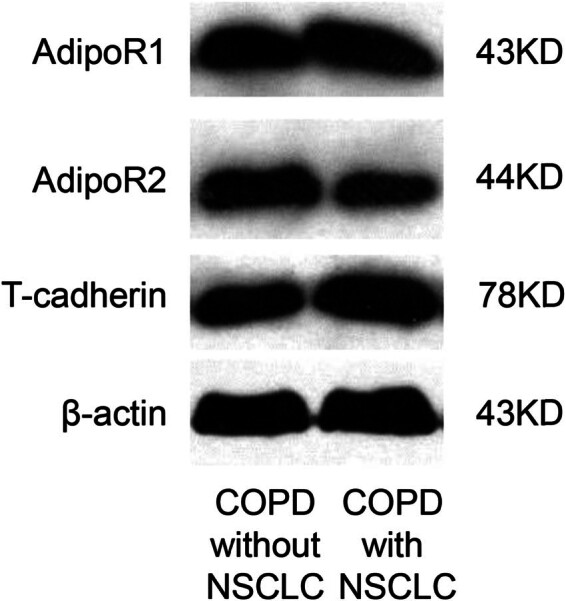
The original bands of adiponectin receptors including AdipoR1, AdipoR2, and T-cadherin in tissues of COPD patients with and without NSCLC detected by the western blot experiment. Abbreviations: AdipoR1, adiponectin receptor 1; AdipoR2, adiponectin receptor 2; COPD, chronic obstructive pulmonary disease; NSCLC, non-small cell lung cancer.

To observe the effects of malignant lesions on these indicators, we further divided patients into those with COPD with NSCLC (*N* = 8) and those with COPD without NSCLC (*N* = 10) groups. Then, we compared their differences in terms of serum and tissue levels of HMW adiponectin, cytokines, and adiponectin receptors. The original bands of T-cadherin, AdipoR1, and AdipoR2 in the tissues detected by western blotting are presented in Figure A2. The results indicated that the HMW adiponectin and cytokine levels in the serum and tissues significantly increased in the COPD with the NSCLC group compared to those in the COPD without the NSCLC group (Table 5).

**Table 5:** Levels of HMW adiponectin, cytokines, and adiponectin receptors in serum and tissues of COPD patients with and without NSCLC

Variables	COPD without NSCLC (*N* = 10)	COPD with NSCLC (*N* = 8)	*t*	*P*
HMW adiponectin (serum, µg/mL)	0.89 ± 0.02	0.99 ± 0.02	−8.816	**<0.01**
HMW adiponectin (tissue, µg/mL)	0.95 ± 0.02	1.03 ± 0.02	−6.236	**<0.01**
IL-6 (serum, pg/mL)	16.90 ± 1.36	24.66 ± 1.27	−9.324	**<0.01**
IL-6 (tissue, pg/mL)	18.74 ± 0.94	25.04 ± 0.69	−12.087	**<0.01**
IL-10 (serum, pg/mL)	3.10 ± 0.19	3.86 ± 0.17	−6.750	**<0.01**
IL-10 (tissue, pg/mL)	3.61 ± 0.26	4.27 ± 0.18	−4.622	**<0.01**
SP-D (serum, ng/mL)	72.08 ± 5.48	89.88 ± 2.25	−6.718	**<0.01**
SP-D (tissue, ng/mL)	80.91 ± 2.61	101.30 ± 4.45	−8.830	**<0.01**
4-HNE (serum, ng/mL)	231.72 ± 11.41	320.11 ± 24.02	−7.431	**<0.01**
4-HNE (tissue, ng/mL)	475.73 ± 34.09	584.93 ± 17.75	−6.353	**<0.01**
TNF-α (serum, pg/mL)	33.30 ± 1.08	39.06 ± 2.06	−9.145	**<0.01**
TNF-α (tissue, pg/mL)	35.96 ± 2.71	44.64 ± 5.11	32.94	**<0.01**
CRP (serum, ng/mL)	194.27 ± 10.12	231.44 ± 41.54	−8.008	**<0.01**
CRP (tissue, ng/mL)	232.43 ± 19.63	343.54 ± 22.29	−8.366	**<0.01**
T-cadherin (serum, ng/mL)	46.26 ± 1.59	52.26 ± 1.710	−5.748	**<0.01**
T-cadherin (tissue)	147.80 ± 25.31	194.60 ± 17.30	−3.413	**<0.05**
AdipoR1 (tissue)	149.8 ± 22.93	185.20 ± 14.97	−2.890	**<0.05**
AdipoR2 (tissue)	161.80 ± 90.11	133.00 ± 10.41	4.676	**<0.01**

HMW: high molecular weight; COPD: chronic obstructive pulmonary disease; NSCLC: non-small-cell lung cancer; IL-6: interleukin-6; IL-10: interleukin-10; SP-D: surfactant protein D; 4-HNE: 4-hydroxynonenal; TNF-α: tumor necrosis factor α; CRP: C reactive protein; AdipoR1: adiponectin receptor 1; AdipoR2: adiponectin receptor 2. Bold *P* < 0.05 indicates statistical significance.

As for adiponectin receptors, the protein expression levels of T-cadherin and AdipoR1 were markedly increased, while AdipoR2 was significantly less expressed in tissues of the COPD with the NSCLC group than in the COPD without the NSCLC group (Table 5).

## 4 Discussion

In this study, we aimed to examine the changes in the levels of HMW adiponectin, adiponectin receptors, and cytokines in patients with COPD. Adiponectin is an adipocyte-specific plasma protein with a molecular weight of 30 kDa and is, therefore, known as Acrp30, whose anti-protease, anti-inflammatory, and antioxidant functions are involved in the pathogenesis of many lung diseases [21]. By comparing the non-COPD and COPD groups, we found that HMW adiponectin was significantly upregulated in the serum and tissues of patients with COPD, in consistency with the results of most studies. Moreover, a meta-analysis suggested that the serum adiponectin concentration in patients with COPD was higher than that in healthy controls [22]. The properties of adiponectin in COPD are regulated by specific receptors that extensively express AdipoR1, AdipoR2, and T-cadherin and are present in epithelial and endothelial lung cells, suggesting a significant role in lung physiology [23]. Thus, the current study further investigated the expression of adiponectin receptors and found that AdipoR1 expression was elevated in COPD tissues and AdipoR2 showed an opposite trend, whereas the T-cadherin levels were not significantly different in either serum or tissues between the COPD and non-COPD groups. Based on immunohistochemical studies of lung sections from patients with COPD and *in vitro* studies of the cell line A549, high expression of adiponectin and AdipoR1, but not AdipoR2, was found [24]. Adiponectin may inhibit the nuclear transactivation of NF-κB via AdipoR1, thereby affecting the viability of A549 cells [25]. T-cadherin can bind HMW adiponectin, which is required for adiponectin to improve vascular tone and reduce inflammation in the lungs [26]. However, little or no T-cadherin expression has been detected in type II bronchiolar alveolar cells of patients with pulmonary alveolar proteinosis [27]. Although no studies have reported the specific expression of T-cadherin in COPD, relevant studies have suggested that T-cadherin is overexpressed in endothelial cells of various tumors [28]. Our study further revealed that the T-cadherin levels were observably higher in the serum and tissues of patients with COPD with NSCLC than in those without NSCLC. T-cadherin may play a crucial role in the progression of cells from an inflammatory state to carcinogenesis without a critical regulatory role in the pre-existing chronic inflammatory response.

To further estimate the role of adiponectin in the regulation of inflammation in COPD, we examined the cytokine levels of patients with COPD and their relationship with adiponectin expression. The results suggested that the levels of IL-6, IL-10, SP-D, 4-HNE, TNF-α, and CRP increased in the serum and tissues of patients with COPD and were prominently positively associated with the expression of HMW adiponectin. However, the anti-inflammatory activity of adiponectin may reduce the production and activity of TNF-α and IL6 in the inflammatory response [29]. Interestingly, 4-HNE can regulate the expression of adiponectin at both the gene and protein levels and can downregulate plasma adiponectin levels associated with obesity [30]. In contrast, our findings suggest that adiponectin may promote the production of IL-6, IL-10, 4-HNE, TNF-α, and CRP in COPD, thereby exerting pro-inflammatory effects. SP-D is a multimeric collection expressed in the lung and non-pulmonary epithelia that exerts antimicrobial effects and inhibits inflammatory responses through a range of host cell responses [31]. The serum SP-D levels have been reported to be higher in patients with pneumonia, tuberculosis, and COPD than in controls [32]. Variables related to increased serum SP-D levels reduce COPD risk and exhibit a decline in lung function [33]. Therefore, we speculated that lipocalin is complex and bidirectional in the inflammatory response to COPD, and it can activate pro-inflammatory mechanisms by upregulating the levels of pro-inflammatory factors, while its anti-inflammatory mechanisms may be largely dependent on regulatory mechanisms with SP-D.

After evaluating the clinical characteristics of COPD, we found no significant differences in the smoking status between the COPD and non-COPD groups, but significant differences were found in PaO_2_, PaCO_2_, and FEV1 (% predicted) levels and CAT score. In clinical practice, these indicators may help clinicians determine the pulmonary and extrapulmonary characteristics of patients and optimize treatment strategies. FEV1 (% predicted) can be used to measure the severity of airflow limitation, CAT score can assess the impact of COPD on patients’ health status, and PaO_2_ can assist in determining whether alveoli are hypoxic and the potential for hypoxemia [34–36]. To further observe the effect of HMW adiponectin on lung function, we evaluated the relationship between HMW adiponectin and these key clinical features and found significant positive correlations between HMW adiponectin levels and FEV1 (% predicted) and CAT score, but a significant negative correlation with PaO_2_. The plasma adiponectin, IL-6, and CRP levels were negatively correlated with FEV1 (% predicted) [37]. In addition, the adiponectin levels increased progressively during COPD disease progression but did not correlate significantly with FEV1 and other inflammatory parameters during the stable phase of COPD [29]. Moreover, FEV1 was significantly reduced at higher adiponectin levels, but this correlation was no longer significant after adjusting for insulin resistance and CRP [38]. Therefore, we can speculate that the effect of adiponectin on lung function is related to insulin resistance and systemic inflammation.

In this study, we found potential associations between HMW adiponectin and cytokine levels and clinical features in COPD; however, these results should be further confirmed using data from a large sample cohort. In future studies, we will delve into the molecular regulatory mechanisms of HMW adiponectin involvement in the inflammatory response in COPD to provide deeper theoretical support for the results of this study.

## 5 Conclusion

HMW adiponectin and cytokine levels were significantly upregulated in patients with COPD compared with those in patients without COPD. AdipoR1 expression increased and AdipoR2 expression decreased in the lung tissue of patients with COPD, whereas T-cadherin expression was not significantly changed. However, after further stratification, T-cadherin expression was significantly upregulated in patients with COPD and NSCLC. In addition, we found significant correlations between the HMW adiponectin and cytokine levels, and the clinical characteristics of COPD.
